# A Learning Strategy Intervention to Promote Self-Regulation, Growth Mindset, and Performance in Introductory Mathematics Courses

**DOI:** 10.3390/ejihpe15100198

**Published:** 2025-09-29

**Authors:** Sayed A. Mostafa, Kalynda Smith, Katrina Nelson, Tamer Elbayoumi, Chinedu Nzekwe

**Affiliations:** 1 Department of Mathematics & Statistics, North Carolina A&T State University, Greensboro, NC 27411, USA; kvnelson@ncat.edu (K.N.); tmelbayoumi@ncat.edu (T.E.); cjnzekwe@ncat.edu (C.N.); 2 Department of Psychology, North Carolina A&T State University, Greensboro, NC 27411, USA; kcsmith7@ncat.edu

**Keywords:** learning strategies, math growth mindset, self-regulated learning, social identities, path analysis

## Abstract

This study investigates the effectiveness of integrating explicit learning-strategy instruction into gatekeeper mathematics courses to foster a math growth mindset, self-regulated learning (SRL), and improved academic performance among underrepresented minority students. The intervention was implemented across four key courses—College Algebra I/II and Calculus I/II—and incorporated evidence-based cognitive, metacognitive, and behavioral learning strategies through course materials, class discussions, and reflective assignments. Grounded in a conceptual framework linking learning-strategy instruction, growth mindset, SRL, and performance—while accounting for students’ social identities—the study explores both direct and indirect effects of the intervention. Using an explanatory sequential mixed-methods design, we first collected quantitative data via pre- and post-surveys/tests and analyzed performance outcomes, followed by qualitative focus groups to contextualize the findings. Results showed no significant effects of the intervention on growth mindset or SRL, nor evidence of mediation through these constructs. The direct effect of the intervention on performance was negative, though baseline mindset, SRL, and pre-course preparedness strongly predicted outcomes. No moderation effects were detected by student identities. The findings suggest that while explicit learning-strategy instruction may not independently shift mindset or SRL in the short term, pre-existing differences in these areas are consequential for performance. Qualitative findings provided further context for understanding how students engaged with the strategies and how instructor implementation shaped outcomes. These insights inform how learning strategies might be more effectively embedded in introductory math to support success and equity in STEM pathways, particularly in post-COVID educational contexts.

## 1. Introduction

Introductory mathematics courses are foundational to STEM education, equipping students with critical quantitative skills necessary for success in the increasingly data-driven STEM disciplines. Performance in these gateway math courses significantly influences students’ transition from high school to college, academic persistence, and graduation outcomes (Carver et al., 2017). Despite their importance, improving student outcomes in these courses remains a persistent challenge—particularly at minority-serving institutions across the United States.

A growing body of research highlights the positive effects of a math growth mindset (GM) and self-regulated learning (SRL) on student achievement and persistence in STEM (Yeager et al., 2019). Similarly, studies have established strong links between explicit instruction in learning strategies and improved academic outcomes (Weinstein et al., 2000; Donker et al., 2014). However, most existing research focuses on K–12 contexts, leaving a critical gap in understanding how such strategies can be effectively applied in postsecondary mathematics education, especially within historically Black colleges and universities (HBCUs).

For example, Karlen et al. (2021) examined how students’ beliefs about the malleability of SRL and their self-concepts influenced academic emotions, awareness of learning strategies, and achievement among Swiss secondary students. Their findings showed that students with growth mindsets and stronger SRL self-concepts experienced more positive emotions, less boredom, greater knowledge of learning strategies, and better academic performance. Gender differences also emerged, with girls reporting stronger SRL self-concepts and greater strategy use, underscoring the value of promoting SRL and GM in diverse educational settings. Building on this, Larson (2023) investigated an SRL intervention infused with GM principles for high school students receiving special education services. While students initially held fixed beliefs about intelligence and self-regulation, post-intervention results revealed a shift toward GM beliefs, though changes in self-regulatory behavior were modest. These findings highlight the complexity of shifting deeply held beliefs about learning and the need for carefully designed interventions.

Given these findings, there is a pressing need to explore whether explicit instruction in cognitive, metacognitive, and management learning strategies can be effectively integrated into introductory college math courses to promote SRL and GM and subsequently enhance performance. This research is particularly relevant for HBCUs, where addressing educational disparities through scalable, evidence-based interventions is of urgent importance.

The present study addresses this gap by implementing a learning strategy intervention across four gateway mathematics courses—College Algebra I/II and Calculus I/II—at an HBCU. The intervention includes targeted course materials, structured classroom discussions, and assignments designed to reinforce SRL and GM principles. We evaluate the effectiveness of this intervention among underrepresented minority students, focusing on its impact on math GM, SRL behaviors, and academic performance. This research is especially timely in the post-COVID educational landscape, where adaptive mindsets and strong self-regulatory skills have become more essential than ever for student success in higher education.

### 1.1. Related Literature

Prevailing beliefs about the fixed nature of mathematical ability have historically hindered student achievement and engagement in mathematics education in the United States (Stevenson et al., 1993). These beliefs, rooted in what Dweck (2000) defines as a fixed mindset, lead students to view intelligence and ability as innate and unchangeable. Such views are especially detrimental in mathematics, a subject often perceived as requiring natural talent rather than effort or practice. This mindset contributes to a persistent pattern of underachievement and disengagement, particularly among students from historically underrepresented groups, including females, African Americans, and Latinx students (Boaler, 2013; Flores, 2007; Sun, 2015). In contrast, a growth mindset—the belief that intelligence and abilities can be developed through effort, effective strategies, and persistence—has been associated with greater resilience in the face of academic challenges and improved learning outcomes (Dweck, 2000).

Another key contributor to academic performance in mathematics is the degree to which students practice self-regulated learning (SRL). SRL encompasses a range of cognitive, metacognitive, motivational, and behavioral processes that learners use to plan, monitor, and control their learning (Zimmerman, 2000). Self-regulated learners actively manage their learning through goal setting, strategy selection, self-monitoring, and self-reflection (Darr & Fisher, 2005). These learners not only persist in challenging tasks but also adjust their approaches based on feedback and task demands. SRL is particularly important in mathematics, a subject that requires flexible thinking, persistence, and the ability to monitor and revise problem-solving strategies (Marchis, 2012; Fauzi & Widjajanti, 2018).

Importantly, the literature suggests that learning strategies play a pivotal role in facilitating both GM and SRL. These strategies serve as the operational bridge between belief systems (e.g., mindset) and self-regulatory behaviors. According to Pressley et al. (1989), learning strategies refer to “processes (or sequences of processes) that, when aligned with the requirements of tasks, enhance performance.” Weinstein et al. (2000) expand this definition by noting that these strategies encompass students’ thoughts, behaviors, and beliefs that facilitate the acquisition, organization, understanding, or application of new knowledge. Examples of such strategies include time management, self-testing, elaboration, organization, and help-seeking behaviors—all of which support students in becoming more autonomous and effective learners. These strategies are not only critical for mastering mathematical content but also reinforce a growth-oriented approach to learning by demonstrating that effortful, strategic engagement leads to success. When explicitly taught and modeled, learning strategies can, thus, promote both a GM and stronger self-regulatory habits, creating a synergistic effect on student motivation and performance.

Across recent studies, GM consistently emerges as an enabler of students’ self-beliefs and self-directed learning, though its effects depend heavily on contextual supports. He and Zhang (2024) demonstrate that GM predicts higher learning self-efficacy, but the strength of this relationship varies with cultural orientation—weakening in settings with greater power distance and uncertainty avoidance, and strengthening under long-term orientation. This suggests that mindset interventions should be paired with structural practices that reduce hierarchy, encourage low-stakes experimentation, and emphasize sustained goal pursuit. Complementing this cultural lens, Jiang et al. (2023) extend the Theory of Planned Behavior to show that GM enhances students’ intentions to self-regulate mainly by shaping positive attitudes toward SRL and improving perceived behavioral control, with teacher support serving as both a key predictor and a modest moderator. Their findings underscore the importance of supportive instructional practices in fostering GM and amplifying SRL intentions. From a broader post-pandemic perspective, Lee and Kwon (2023) highlight the interplay among GM, SRL, well-being, and smart-device utilization. They find that purposeful use of technology and blended course designs can promote motivation and engagement when integrated with community building and strong instructor presence. In another recent study, Wang et al. (2023) validated a multidimensional model of self-regulated writing strategies among advanced English as a Foreign Language (EFL) learners, showing that motivational regulation, cognition/metacognition, and social behavior are distinct but interrelated components. Their results support the notion that SRL is not monolithic, and interventions aiming to reshape learning strategies should attend not just to which strategies are taught, but how they are measured, scaffolded, and contextualized. Together, these contributions confirm GM’s central role in learning but also emphasize that its benefits are contingent on cultural, motivational, instructional, and measurement conditions. They collectively call for longitudinal and multi-context studies to move beyond single-site, cross-sectional, self-report designs.

Despite growing recognition of the value of mindset and SRL, much of the existing literature focuses on K–12 settings, with relatively little research investigating the integration of these constructs into college-level mathematics instruction, particularly at HBCUs and other minority-serving institutions. This gap presents an opportunity to explore how targeted instructional interventions—centered on learning strategies—can promote GM and SRL in postsecondary math contexts, thereby supporting academic success and reducing achievement gaps.

### 1.2. Conceptual Framework and Research Questions

Our proposed conceptual framework is poised to serve as a model for future research examining the intricate connections among instructional strategies for learning, the development of a math GM, SRL, and math performance. This framework postulates the following hypotheses: (i) Learning-strategy instruction has a direct effect on students’ performance (Donker et al., 2014); (ii) learning-strategy instruction can indirectly influence performance by fostering students’ math GM, leading them to perceive new avenues for growth in learning and achievement, and students’ SRL (McDaniel & Einstein, 2020); and (iii) students’ various social identities (such as racial, gender, and math identities) are likely to moderate the relationships described in (i)-(ii).

**Figure 1:**
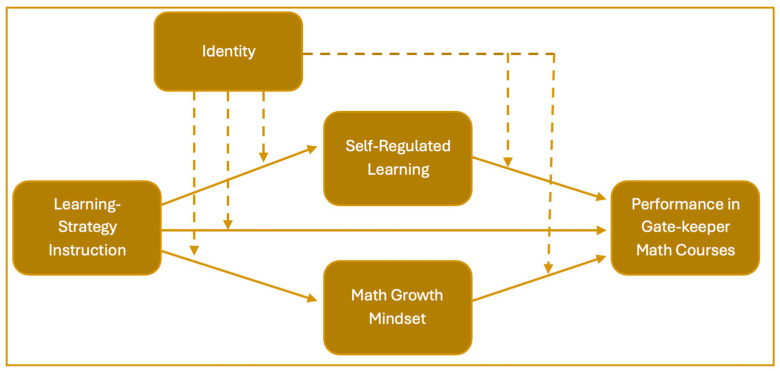
Conceptual framework.

Our proposed conceptual framework (see Figure 1) is designed to guide future research investigating the mechanisms by which learning-strategy instruction affects student outcomes in introductory mathematics. Specifically, it models the interrelationships among explicit instruction in cognitive and metacognitive learning strategies, the development of self-regulated learning (SRL), math GM, academic performance, and identity-related factors.

This framework posits three primary hypotheses:

1. Direct Effects: Learning-strategy instruction has a direct positive effect on students’ academic performance in gatekeeper mathematics courses. This aligns with prior evidence suggesting that explicit strategy instruction enhances students’ ability to manage their learning processes and apply effective problem-solving techniques (Donker et al., 2014).
2. Indirect Effects via GM and SRL: Learning-strategy instruction also influences performance indirectly, by fostering both a math GM and self-regulated learning. When students are taught how to learn—through strategies such as goal setting, self-monitoring, and reflection—they begin to see intelligence and mathematical ability as malleable rather than fixed. This mindset shift encourages persistence and adaptive learning behaviors (McDaniel & Einstein, 2020), which in turn contribute to improved academic performance.
3. Moderating Role of Identity: Students’ social identities—including racial, gender, and math identities—may moderate these relationships. For example, a student who strongly identifies with mathematics may be more responsive to learning-strategy instruction, whereas students from groups historically underrepresented in math may require more intentional efforts to build a GM and SRL. Identity also shapes how students interpret failure, seek help, and persist through difficulty, all of which influence the effectiveness of self-regulation and mindset interventions.

As shown in the framework, identity is not just a background variable but an active force that shapes how students engage with instructional strategies and internalize growth-oriented beliefs. The model includes both solid arrows (representing primary hypothesized pathways) and dashed arrows (representing moderating influences), reflecting the dynamic nature of these constructs in shaping students’ learning experiences and performance outcomes.

By integrating instructional, psychological, and sociocultural components, this framework highlights the multi-level and interactive processes that contribute to student success in introductory mathematics. It underscores the importance of not only teaching content but also fostering the beliefs, strategies, and identities that enable all students—particularly those from underrepresented backgrounds—to thrive in introductory mathematics courses.

Guided by the above framework, this study addresses the following three *research questions:*

**RQ1.** To what extent does learning-strategy instruction in gatekeeper courses promote a math growth mindset (GM)?
**RQ2.** To what extent does learning-strategy instruction in gatekeeper math courses promote self-regulated learning (SRL)?
**RQ3.** To what extent do learning-strategy instruction, math GM, and SRL predict students’ performance in gatekeeper math courses?

## 2. Materials and Methods

An explanatory sequential mixed-methods design was employed to collect data and address the research questions. In Phase 1, quantitative data were collected via surveys and analyzed using several inferential analyses. In Phase 2, a focus group protocol was created based on the quantitative findings to assist in interpreting those findings. Phase 2 data was analyzed using qualitative coding and thematic analysis. These phases are described in detail below.

### 2.1. Intervention

To promote both a math GM and self-regulated learning (SRL), we embedded learning strategies directly into four gatekeeper mathematics courses through a variety of instructional activities. The intervention focused on four key strategies: elaboration (cognitive), self-testing and adaptation of learning approach (metacognitive), and effort management (behavioral/management).

Elaboration strategies help students form meaningful connections between new material and prior knowledge. Donker et al. (2014) found such strategies to be particularly effective in mathematics learning. Instructors supported elaboration by prompting students to explain their reasoning, justify their solutions, and engage in sense-making during class discussions and discussion board assignments. These activities align with Zimmerman’s (2000) self-reflection phase of SRL and foster a math GM by encouraging deeper cognitive engagement (e.g., Sun, 2015).

Self-testing and adaptive learning strategies, which span the performance and reflection phases of SRL, were integrated through frequent formative assessments and opportunities for multiple attempts. These practices promote self-monitoring and reflection while reinforcing GM beliefs by emphasizing learning through persistence and revision (Sun, 2015). Additionally, math tasks with multiple solution paths communicated that ability can be developed, supporting both SRL and GM development.

Effort management strategies were encouraged through effort-focused feedback, which aligns with the forethought phase of SRL. By attributing mathematical success to effort and strategy use rather than innate ability, instructors helped students internalize adaptive beliefs about learning and persistence.

Students were exposed to these learning strategies through a sequence of online discussion board assignments and reflective in-class activities. For example, in Week 3, students watched a video on the study cycle and contributed posts and replies in an online forum. The following week, the instructor revisited the study cycle in the context of course content, reinforcing students’ earlier reflections and providing practical application.

Table 1 summarizes the intervention activities. Appendix A provides a detailed breakdown of the learning strategy instruction, including implementation examples and associated student learning outcomes.

### 2.2. Quantitative Study

The quantitative study utilizes a repeated-measures between-subjects design where four sections in each of the four target math courses (College Algebra I/II and Calculus I/II) at a large HBCU were assigned to a treatment group (2 sections taught by 2 different instructors) or a control group (2 sections taught by the same two treatment instructors). Treatment students are taught about effective math learning strategies, including elaboration, self-testing, effort and time management, and test-taking strategies in the form of class discussions and activities, discussion board assignments, and short videos and quizzes. Control students, on the other hand, are taught the same course content without any instruction on learning strategies.

#### 2.2.1. Randomization, Data, and Scales

To evaluate the effectiveness of integrating learning strategies into gatekeeper math courses, we use data collected during the 2022–2023 academic year from students at the study institution. The data come from 32 sections (16 treatment and 16 control) across four introductory math courses. In each semester (Fall and Spring), 8 instructors each taught one treatment and one control section. Some instructors participated in both semesters, while others taught in only one. The assignment of sections to control and treatment conditions was designed to balance class meeting times across sections. Assigning sections to the conditions was implemented by a study coordinator not involved in the delivery of instruction. We monitored fidelity and exposure informally through weekly reminders and instructor check-ins to ensure delivery of the core components (explicit strategy instruction, metacognitive prompts, and assessment linkage). We also collected student attendance logs for each section. The data consists of (i) students’ responses to pre- and post-surveys about their math mindset, SRL, and math, gender, and racial identities; (ii) students’ scores on pre- and post-tests related to course content; and (iii) students’ demographic (gender, PELL eligibility status, rurality, and residency) and academic (STEM status, classification, attendance, and overall course grade) characteristics. A total of 485 students (230 control and 255 treatment) completed the pre- and post-surveys, and spent at least 5 min on each. All analyses in this study were conducted under an intent-to-treat framework.

Hocker’s (2017) modified math mindset scale was adapted and used for measuring students’ math mindsets. The original scale has 16 items that address multiple domains: math mindset, beliefs about the nature of math, mastery/performance orientation, and identification with math. Only the items related to the math mindset were used. Students answered items on a 6-point Likert scale, ranging from 1 = “Strongly disagree” to 6 = “Strongly agree”. The Self-Regulation Strategy Inventory–Self-Report, developed by Cleary (2006), was used to measure students’ SRL. The original SRL scale, validated on a sample of high school students, had 28 items divided into three subscales: (a) Managing Environment and Behavior, (b) Maladaptive Regulatory Behaviors, and (c) Seeking and Learning Information. Students answered the remaining 20 items on a 5-point Likert scale, ranging from 1 = “almost never” to 5 = “almost always”. Racial identity was measured using Sellers et al.’s (1997) Multidimensional Inventory of Black Identity (MIBI) for Black students and Brown et al.’s (2014) Multigroup Ethnic Identity Measure (MEIM) for non-Black students. Gender identity was measured using a modified version of the MIBI scale. Finally, math identity was measured using Lock et al.’s (2013) math identity scale, which consists of three subscales: (a) math competency, (b) math recognition, and (c) math interest. Please refer to Appendix B, Appendix C, Appendix D, Appendix E and Appendix F for the list of items on each scale and to Section 3 for the psychometric statistics.

#### 2.2.2. Quantitative Analysis

The quantitative data analysis included descriptive statistics, psychometric analyses of the mindset, SRL, and various identity scales, correlations, and regression modeling (path analysis) to test the theoretical framework in Figure 1 and address the research questions RQ1–RQ3. We use the following notations in the analysis:

- $GM_{i,\mathrm{post}}$($GM_{i,\mathrm{pre}})$: post-test (pre-test) growth mindset score for student i
- $SRL_{i,\mathrm{post}}$($SRL_{i,\mathrm{pre}})$: post-test (pre-test) SRL score for student i
- $Y_{i,\;post}$($Y_{i,\;pre}$): post-test (pre-test) performance score for student i
- $\Delta GM_{i}=GM_{i,\mathrm{post}}-GM_{i,\mathrm{pre}}$: change in GM for student i
- $\Delta SRL_{i}=SRL_{i,\mathrm{post}}-SRL_{i,\mathrm{pre}}$: change in SRL for student i
- $\Delta Y_{i}=Y_{i,\mathrm{post}}-Y_{i,\mathrm{pre}}$: change in performance score for student i
- $T_{i}$ = treatment (learning strategy instruction) indicator
- $ID_{i}$ = identity (math, gender, and racial) moderators
- $X_{i}$ = pre-specified covariates (including students’ demographic and academic characteristics and instructional context, i.e., course).

##### Path Analysis Models

Model 1. Treatment → Growth Mindset ($a_{1}$ path)

$$GM_{post,i}=\alpha_{0}+a_{1}T_{i}+\alpha_{1}GM_{pre,i}+\alpha_{2}SRL_{pre,i}+\alpha_{3}Y_{pre,i}+\alpha_{4}^{T}{ID}_{i}+\alpha_{5}^{T}X_{i}+\varepsilon_{i}^{GM}$$

Model 2. Treatment → Self-Regulated Learning ($a_{2}$ path)

$$SRL_{post,i}=\beta_{0}+a_{2}T_{i}+\beta_{1}GM_{pre,i}+\beta_{2}SRL_{pre,i}+\beta_{3}Y_{pre,i}+\beta_{4}^{T}{ID}_{i}+\beta_{5}^{T}X_{i}+\varepsilon_{i}^{SRL}$$

Model 3. Outcome on Treatment, GM, and SRL ($c^{\prime }$, $b_{1}$, and $b_{2}$ paths)

$$Y_{post,i}=\gamma_{0}+c^{\prime }T_{i}+b_{1}GM_{post,i}+b_{2}SRL_{post,i}+\gamma_{1}GM_{pre,i}+\gamma_{2}SRL_{pre,i}+\gamma_{3}Y_{pre,i}\;+\gamma_{4}^{T}{ID}_{i}+\gamma_{5}^{T}X_{i}+\varepsilon_{i}^{Y}$$

Model 4. Moderation of Treatment → GM (identity moderates the $a_{1}$ path)

$$GM_{post,i}=\delta_{0}+a_{1}T_{i}+\delta_{1}ID_{i}+\delta_{2}(T_{i}\times ID_{i})+\delta_{3}GM_{pre,i}+\delta_{4}SRL_{pre,i}+\delta_{5}Y_{pre,i}+\delta_{6}^{T}X_{i}+\varepsilon_{i}^{GM}$$

Model 5. Moderation of Treatment → SRL (identity moderates the $a_{2}$ path)

$$SRL_{post,i}=\theta_{0}+a_{2}T_{i}+\theta_{1}ID_{i}+\theta_{2}(T_{i}\times ID_{i})+\theta_{3}GM_{pre,i}+\theta_{4}SRL_{pre,i}+\theta_{5}Y_{pre,i}+\theta_{6}^{T}X_{i}+\varepsilon_{i}^{SRL}$$

Model 6. Moderation of Treatment/Mediator → Outcome (identity moderates the $c^{\prime }$, $b_{1}$, and $b_{2}$ paths)

$$\begin{array}{cc} Y_{post,i} & =\kappa_{0}+c^{\prime }T_{i}+b_{1}GM_{post,i}+b_{2}SRL_{post,i}+\kappa_{1}ID_{i}\\ & +\kappa_{2}(T_{i}\times ID_{i})+\kappa_{3}(GM_{post,i}\times ID_{i})+\kappa_{4}(SRL_{post,i}\times ID_{i})\\ & +\kappa_{5}GM_{pre,i}+\kappa_{6}SRL_{pre,i}+\kappa_{7}Y_{pre,i}+\kappa_{8}^{T}X_{i}+\varepsilon_{i}^{Y} \end{array}$$

All analyses were conducted using the open-source statistical software R version 4.4.2 (R Core Team, 2024). A 5% significance level is used throughout the study.

### 2.3. Qualitative Study

#### 2.3.1. Participants

Participants were 24 freshmen, 8 sophomores, and 7 juniors from diverse majors, including Animal Science, Atmospheric Science, Biology, Business, Chemistry, Computer Science, Engineering, Economics, Graphic Design, Health Services Management, Kinesiology, Marketing, Mathematics, and Physics. Participants were recruited from College Algebra I, College Algebra II, Calculus I, and Calculus II. There were 10 focus groups and two interviews.

#### 2.3.2. Procedures

Participants were recruited to participate in focus groups via email during the semester post-course completion. When participants responded to a scheduling poll, focus groups or interviews were scheduled based on students’ availability, course, and study conditions. When beginning focus groups, participants completed an Informed Consent Form and were then asked to introduce themselves. Participants were then interviewed by a Co-PI and asked several questions from a focus group protocol designed to address the research questions. First, participants were asked questions regarding their experiences with math, in general, to create a rapport with participants and give some context to their attitudes about math and their experiences in the specific math course in which they were enrolled the previous semester to address the research questions and finally questions regarding their perceptions of the intersection of their personal identity(ies) and their math experiences. The questions can be found in Appendix G. The focus groups lasted approximately one hour and were tape-recorded. When the focus group session ended, participants were thanked for their time. Participants were compensated with $25 VISA gift cards.

#### 2.3.3. Qualitative Analysis

The audio recordings were transcribed by a third-party vendor and coded using deidentified data by one of the authors and an undergraduate student who was trained in qualitative data analysis. Both inductive and deductive coding approaches were used: inductive coding allowed themes to emerge naturally from the raw data, while deductive coding was guided by the focus group protocol questions. A deductive thematic analysis was then conducted to identify patterns that could further support and contextualize the quantitative findings.

## 3. Results

In this section, we present the results of both the quantitative and qualitative analyses.

### 3.1. Quantitative Results

Table 2 presents descriptive statistics for students in the control and treatment sections. Overall, the two groups appear reasonably comparable in terms of background characteristics.

We first summarize the results of the psychometric analyses conducted to compute the various constructs of interest from the survey data items. These analyses consisted of exploratory factor analysis using the “fa()” function in the “semTools” R package ver. 0.5-7 (Jorgensen et al., 2025) and calculation of the McDonald’s *coefficient omega* of internal reliability (McDonald, 1999; Flora, 2020) computed using the “cfa()” function in the “lavaan” R package ver. 0.6-19 (Rosseel, 2012). The factor analysis of the mindset items showed a good fit for the mindset scale to the data, with the root mean square of the residuals (*RMSR*) = 0.003/0.002 and *Omega* = 0.69/0.73 for pre-survey/post-survey. On the other hand, the SRL items did not fit the original three-factor structure, with the items of the Seeking and Learning Information subscale not loading on a single factor as hypothesized. The two-factor structure provided an acceptable fit, with the Managing Environment and Behavior (*SRL-1*) and the Maladaptive Regulatory Behaviors (*SRL-2*) subscales forming two separate factors (presurvey *RMSR* = 0.057 and *Omega* = 0.84 & post-survey *RMSR* = 0.063 and 0.83). These two subscales did not load on a common factor (pre-survey factor Pearson correlation coefficient *r* = 0.032 & post-survey *r* = −0.068) and were analyzed separately. The math, gender, and racial identity scales demonstrated an acceptable fit to the data, as indicated by the fit statistics (RSMR and Omega coefficient) in Table 3. Math identity was composed of three subscales: competency, recognition, and interest, and they were kept separate in the analysis. Gender identity had two separate subscales: centrality and reflection. For racial identity, the scale for Black students—MIBI—had three subscales (centrality, private regard, and public regard), whereas the scale for non-Black students—MEIM—had two subscales (exploration and commitment). To facilitate calculating a common racial identity variable for both groups, the MIBI subscales were combined into one composite, and similarly, the MEIM subscales were combined into one composite.

Table 4 presents pre-, post-, and difference scores for math mindset and self-regulated learning (SRL) across the control (CTRL) and treatment (TRT) groups. Both cohorts showed marginal, statistically indistinguishable declines in mindset (CTRL = −0.20; TRT = −0.18): the control mean reduced from 4.47 (*SD* = 0.90) to 4.27 (*SD* = 0.96), whereas the treatment mean moved from 4.42 (*SD* = 0.98) to 4.25 (*SD* = 1.04). SRL results were similar. For SRL-1, which captures management of environment and behavior, CTRL edged up from 3.43 (*SD* = 0.67) to 3.45 (*SD* = 0.63) for a +0.03 gain (*SD* = 0.53), while TRT rose from 3.41 (*SD* = 0.66) to 3.45 (*SD* = 0.66) for a +0.04 gain (*SD* = 0.50). Conversely, SRL-2, where higher scores denote fewer maladaptive strategies, decreased slightly in both groups (CTRL = −0.16; TRT = −0.21), suggesting possible challenges in curbing ineffective learning habits despite the intervention.

**Figure 2:**
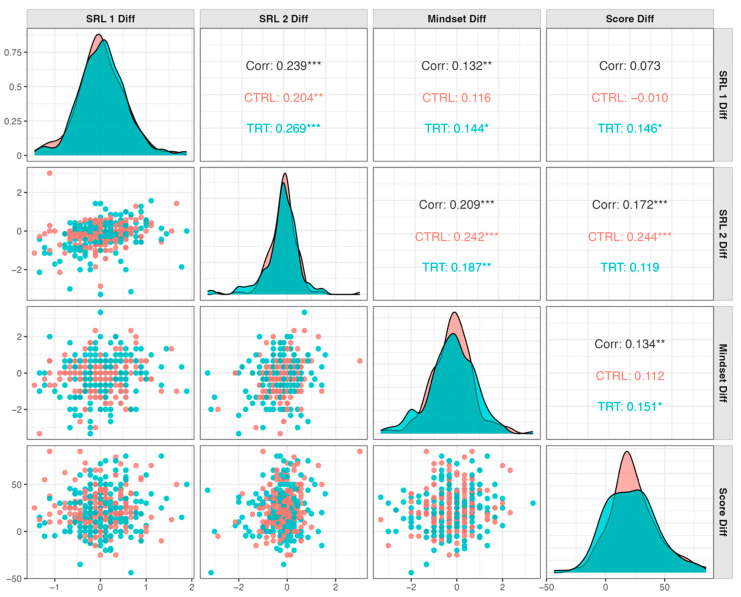
Correlation of pre-to-post differences in SRL, GM, and performance grouped by intervention. Significance codes: ‘***’ ≡ *p*-value < 0.001; ‘**’ ≡ *p*-value < 0.01; ‘*’ ≡ *p*-value < 0.05.

Correlation testing, as shown in Figure 2, mapped how the intervention’s psychological and behavioral shifts were intertwined with Research Questions 1 and 2, revealing a network of modest yet meaningful associations. Change scores on the two self-regulation dimensions rose and fell together (SRL-1 Diff × SRL-2 Diff: *r* = 0.239, *p* < 0.001), an alignment that grew stronger among students who received explicit learning-strategy instruction (TRT: *r* = 0.269 vs. CTRL: *r* = 0.204), implying the instructional scaffold promoted parallel refinement of adaptive habits and reduction of maladaptive ones. Concurrently, mindset shifts displayed small but positive ties to shifts on both SRL facets (Mindset Diff × SRL-1 Diff: *r* = 0.132; Mindset Diff × SRL-2 Diff: *r* = 0.209), with the association between mindset shifts and SRL-1 shifts being statistically significant only among the treatment cohort whereas the association with SRL-2 shifts was statistically significant across both control and treatment groups. This suggests that as students adjusted their regulatory repertoire, they also nudged their beliefs toward a growth orientation. Performance gains, though weaker, trended in a similar direction: score change was positively correlated with mindset shifts (*r* = 0.134) and SRL-1 shifts (*r* = 0.073), inching higher and being statistically significant only in the treatment group (0.151 and 0.146). A stronger correlation was observed between score change and SRL-2 shifts (*r* = 0.172), but it was only significant among control students (*r* = 0.244). A deeper Pearson analysis confirmed that starting dispositions carried weight such that initial and post-semester scores were moderately, but significantly, linked for mindset (*r* = 0.51), SRL-1 (*r* = 0.69), and SRL-2 (*r* = 0.47) yet it also showed that post-semester mindset bore only low insignificant ties to early environment and behaviors regulation (*r* = −0.03) and early maladaptive regulation (*r* = 0.33). Collectively, these results suggest that while early traits shape future outcomes, targeted strategy instruction can promote gradual, coordinated improvements in both mindset and self-regulation.

Path Analysis (Mediation) Results:

Results from the path models are presented in Table 5. Treatment assignment was not significantly associated with post-test GM (a_1_: β = −0.046, *p* = 0.599), SRL1 (a_2_: β = −0.006, *p* = 0.908), or SRL2 (a_2’_: β = −0.082, *p* = 0.178). Likewise, the post-test mediators were not significant predictors of post-test performance: GM (b_1_: β = 0.887, *p* = 0.366), SRL1 (b_2_: β = 0.367, *p* = 0.832), or SRL2 (b_2’_: β = 2.308, *p* = 0.102).

By contrast, the direct effect of treatment on post-test performance remained significant after adjusting for the mediators (c′: β = −3.830, *p* = 0.016), suggesting that treatment was directly associated with lower performance outcomes.

Table 6 shows the decomposition of the total effect. None of the indirect effects through GM (a_1_ × b_1_), SRL1 (a_2_ × b_2_), or SRL2 (a_2’_ × b_2’_) were statistically significant, and the sum of all indirect effects was small (β = −0.232, 95% CI [−0.984, 0.143]). The total effect of treatment on performance was significant (β = −4.061, 95% CI [−6.945, −0.947]), with only 5.7% of this effect accounted for by the mediators.

Overall, the mediation models provide little evidence that GM or self-regulated learning served as pathways through which the treatment influenced performance. Instead, the treatment effect appears to operate primarily through a direct path.

Moderation Results:

To test whether the treatment effects varied by student identity measures, we estimated the moderation models 4–6, including interaction terms between treatment (role) and math, gender, and racial identity variables. Wald χ^2^ tests of the joint null hypothesis that all interaction coefficients equal zero were not significant in any model (all *p* > 0.05). This indicates that the treatment effects did not significantly differ across levels of students’ math, gender, or racial identity measures. In other words, there was no evidence that identity moderated the effects of treatment on GM, SRL, or subsequent performance. Including interaction terms did not improve model fit compared to models with only main effects. Table 7 below summarizes the results of the models with only main effects.

Consistent with the earlier path analyses (Table 5 and Table 6), the results in Table 7 indicate that treatment assignment was not significantly related to post-test GM (β = −0.046, *p* > 0.10), SRL1 (β = −0.006, *p* > 0.10), or SRL2 (β = −0.082, *p* > 0.10), providing little evidence that the learning-strategy instruction directly improved students’ GM or self-regulated learning. This addresses **RQ1** and **RQ2**, suggesting that the intervention did not produce measurable gains in these psychosocial and learning-strategy constructs.

In contrast, several baseline covariates strongly predicted post-test outcomes. For example, prior mindset was positively associated with the post-test mindset (β = 0.464, *p* < 0.001), while $SRL1_{pre}$ (β = 0.656, *p* < 0.001) and $SRL2_{pre}$ (β = 0.459, *p* < 0.001) were the strongest predictors of their respective post-test measures. This underscores the stability of these constructs over time.

Turning to **RQ3**, the performance model shows that treatment had a significant negative direct effect on post-content-test performance (β = −3.830, *p* < 0.05), even after accounting for post-test mindset and SRL measures. None of the mediators significantly predicted post-test performance, consistent with the indirect effects reported in Table 6. Instead, post-test performance was more strongly explained by academic background (e.g., pre-test score, β = 0.118, *p* < 0.05), course enrollment (e.g., Calculus I: β = −18.865, *p* < 0.001), and overall course grade (e.g., C grade: β = −18.173, *p* < 0.001). Socio-demographic covariates such as PELL eligibility (β = 6.322, *p* < 0.05) also emerged as significant predictors.

In all models, the low proportion of variance explained by the model (adjusted R^2^) suggests that additional factors other than those included in the models might be driving changes in GM, SRL, and performance.

Overall, the combined results from Table 5, Table 6 and Table 7 show that while the intervention did not foster measurable improvements in GM or SRL, treatment had a direct negative association with post-content-test performance. Performance outcomes were shaped more by baseline academic standing, pre-course readiness, instructional context (e.g., Algebra vs. Calculus course), and demographic factors.

### 3.2. Qualitative Results

In this section, we report the results of the qualitative analysis. First, we summarize the focus group participation in Table 8. A sample of the focus group questions and corresponding codes is given in Table 9; please refer to Appendix G for the full list of focus group questions.

Students were asked how their course differed from previous courses to determine how they responded to the discussion boards more organically, but if students in the treatment condition did not report out about the discussion boards, they were asked about them directly to help determine students’ perceptions of the treatment. In a calculus focus group, five students reported they did not have discussion boards when they did, in another calculus focus group two students did not mention the discussion boards, although they had them. The remaining focus groups and interviews remembered doing the discussion boards, but four reported that doing these boards did not change their behaviors. A student in Calculus II said, “*I know a lot of the students didn’t actually look at that. They just kinda glanced over it and answered the questions, and then moved on*.”

A student in Algebra II was able to give a more detailed description of the treatment activities:

*Most of the discussion boards just ranged on ways to better your study habits, so we just watched a bunch of videos on different ways you could do so, … like reaching out on a textbook or peer tutoring or finding your [own private] tutor, and … what steps you would take to get [one]. I wanna say the discussion boards for me weren’t [very] helpful because I already kinda knew those techniques, but I guess for people that don’t know. I guess that would be helpful for them…*

Of the four that said they changed their behavior as a result of the discussion boards, they all referenced their study habits. A student in Algebra explained:

*Yeah, I feel like I changed my behavior at the beginning of the semester. … I would see … my grades … weren’t what I needed them to be. So, I feel like I kinda … took that into consideration when he said … studying it after class [is helpful]. … [F]or my class, there [were] office hours… right after our class, so I would go like right after class. … [A]nd it would help me because … we would talk about previous classes or … previous [topics], then we would talk about what we learned … in this class. So, it helped me [be] much better.*

#### Themes

Several key themes emerged that directly address the research questions and provide deeper insight into the quantitative findings. These themes include *Math Mindset, Self-Regulated Learning,* and *the professor’s impact*.

Math Mindset

To assess students’ math mindsets, they were asked a series of reflective questions. First, they were prompted to consider their general experiences with math before and during college: “*To what degree do you feel your math abilities have changed over time?*” This question was then repeated in the context of their current math course. Students were also asked: “*Do you think your math abilities can change over time?*” followed by a related question: “*Have you always thought this way?*”

In response, 34 students indicated that their math abilities had grown or could grow, with one student responding, “kind of.” Six students specifically noted that their math skills had improved. Notably, no students stated that their abilities could not change. These findings suggest that, across both control and treatment groups and across various courses, students generally held a GM.

A student in the control group of an Algebra II course shared:

*I think just like how it can improve, it can also decrease, so it’s something I always try to stay on top of. Like during the summer, I am going to those channels I usually use during the school year. I’m watching … trying to get a little bit ahead of reviewing stuff that …we may go over the next year, so when we get to those subjects, I’m kinda familiar with it*.

(codes: math GM, math preparation)

Similarly, students in the treatment condition of an Algebra course said:

*Yes. I don’t think that anyone … is just incapable of … − not capable of like being good at math. I feel like … it’s just something that you have to put your mind to. … Not everybody is really good at math, so it’s kinda like you have to … strive to do better in math*.

(codes: math ability, math GM)

*“Yeah, I think anything you put effort in and practice will be better even if you feel like you’re trash at it. Like repetition and just being yourself grace will help in any subject and stuff.”*.

(codes: math GM)

In an Algebra II course under the treatment condition, another student reflected on their progress:

*I definitely think it’s grown. I was able to do more studying this year than I did before … I was just … not studying and thinking that I could just get it just like that when I wasn’t able to. And so now, when it comes to more formulas for … this specific math, … you have to study because you’re not gonna be able to get it at all unless you take your time to actually do it and not … give up on yourself either*.

(codes: math GM, math preparation)

However, not all students reported recent improvements. Some shared that their math abilities had declined, often citing the challenges of college-level coursework or the lasting effects of the COVID-19 pandemic

For example, a student in the control condition of an Algebra class stated:

*… I was actually always advanced in math. I was actually really good at math. I was great at math. I was always ahead in math, but then after COVID, I got so behind. My brain was just all messed up. It got really bad, and I’m still trying to get myself there. So yeah, it’s just bad*.

(codes: COVID impact, math ability)

Another student stated:

*“I’d probably say decrease all things considered. A lot more of things you have to remember now. The whole basis of it has not really changed at all but it’s just … is more stuff stacked on top of each other and a bunch of rules that coincide.*

(codes: math ability)

Self-Regulated Learning (SRL)

Students were asked a series of questions to determine whether they engaged in self-regulated learning, and responses indicated that such behaviors were present across all courses and conditions. When asked, “When are you most engaged in math?”, students identified a range of activities that helped them stay focused and involved. Seven students reported being most engaged when working on practice problems, while six said they were most engaged during group work. Four students found homework to be the most engaging, and individual responses included using internet videos, the Pearson online platform provided with the course, and reviewing personal notes. These varied responses highlight the diverse strategies students use to take ownership of their learning.

When asked about their ideal way of learning a new math concept, the majority of students—23 in total—indicated a preference for learning outside of class. Among these, 12 students favored doing practice problems, five preferred using internet videos, and another five relied on reviewing their class notes. One student reported group work as their preferred method. Additionally, two students expressed a preference for receiving study notes directly from the professor.

A student in the control group of a Calculus course described their approach:

*“Yeah. Usually just watching [YouTube] videos, paraphrasing those videos, paraphrasing notes as well. Doing some example problems really helps”*.

(codes: math learning strategies)

When asked, *“What types of tasks did you do to help you learn math?”*, students across all focus groups reported using self-regulated resources—both those provided by the course and external tools like internet videos.

For example, a student in the control group of a College Algebra course shared:

*“I would say the Help Me Solve on Pearson, that saved me a lot of going through YouTube videos and looking up formulas and how to complete formulas. So I would say the Help Me Solve because it was straight to the point and it tells you how to get it done in the correct way”*.

(codes: Pearson experience, math learning strategies)

Another student in the same course mentioned using Photomath:

*Sometimes I use the Photomath to get steps of something, ‘cause I don’t like the steps Pearson gives me, so I would use [Photomath] to see how they did it. Usually … I get a better understanding with how they did it. …*.

(codes: Pearson experience, math learning strategies)

Students in the treatment group also referenced external resources:

*Yeah, Khan Academy definitely has … good videos … teaching … different methods because everybody doesn’t teach math the same, so I wouldn’t say there’s a specific way to teach math. It’s like whatever you understand, you’re probably gonna teach. So if you don’t understand it, I’m sure [there’re] … other methods to like learn the same exact part of the curriculum*.

(codes: math learning strategies)

*“I would Google practice problems on the topic and like go through and do all of them just to make sure that I had it down.”*.

(codes: math learning strategies)

Instructor Impact

Notably, many students indicated that their perceived math ability was closely tied to their professor’s teaching ability or instructional style. This suggests that some students may not have felt a strong sense of personal agency over their own learning.

As one student in the treatment group of a Calculus course explained:

*I think your instructor can 100 percent influence your abilities, because I think you could be good at math but if you have a bad instructor, it will make you think you’re bad at it, so you might … turn away from it and—yeah…*.

(codes: negative professor impact, math ability)

A student in the control group of an Algebra course echoed this sentiment:

*[W]hen it comes down to the professor because I’ve had great math teachers where they teach, and I automatically understand what’s happening. And then I get to college and it’s like I’m dealing with people who feel like they don’t have the time to spare to help you or help you understand what’s being given. And it’s frustrating …*.

(code: positive professor impact, negative professor impact)

Another student in the same course emphasized how both the pace and approach of the professor affected their experience:

*I feel like in general I do pretty well in math. Though kind of like the previous questions, it really kinda depends on how good or bad the teacher is, how fast they go, how willing they are to slow down or reteach. Like I said, it—math, it isn’t really like a subject where you’re either good at it or you’re not. It’s really just whether or not you’re given a good space or ways to figure it out…*.

(codes: math ability, professor impact, math GM)

Similarly, a student in the control group of an advanced Algebra course attributed their confidence in math directly to their professor:

*I say I have high ability. I think that—the only reason is because of the professor. Like I said, I think the professor is very important. She’ll help you understand it and get through it or not. … The professor will help you understand it and get through it and just use your time more wisely. I feel like if you just get lost in math, you’ll just spend hours on it and then just give up at the end of the day*.

(codes: math ability, professor impact, math GM)

Despite placing significant importance on their professors for success, few students reported taking advantage of office hours or seeking help outside of class. One student in the treatment group of an advanced Calculus course reflected:

*That would also be one thing I need to be cognizant of, because I’ve been told multiple times that it’s better to just—if you don’t understand something, just go to your professor in office hours, but like me, many students still don’t do that and it’s kinda just—it just seems awkward in a way, but yeah—I’m not sure how to explain it. I just know I don’t really do the things that I know I’ve been told will—that have been recommended that I do*.

(code: resource utilization)

## 4. Discussion

The results of this study provide nuanced insight into how explicit learning-strategy instruction relates to students’ beliefs about mathematics and their self-regulatory behaviors in introductory college math courses at a minority-serving institution, particularly an HBCU. While the intervention did not produce statistically significant changes in math growth mindset (GM) or self-regulated learning (SRL), the observed positive—though nonsignificant—associations between improvements in these domains and performance are consistent with prior research linking GM and SRL to achievement outcomes.

Considering earlier work, the modest quantitative effects align with Larson’s (2023) conclusion that shifting deeply held beliefs requires sustained or intensive engagement. Karlen et al. (2021) found that students with stronger SRL self-concepts and growth mindsets reported more positive emotions and higher achievement, but the present results suggest that such relationships may not manifest as clearly in short-term, course-bound interventions, particularly when prior beliefs and habits are well established. The lack of significant effects also echoes Jiang et al.’s (2023) findings that teacher support is a critical moderator for translating GM into SRL intentions, pointing to the importance of structural and instructional conditions in shaping how such relationships unfold.

The qualitative findings further echo Dweck’s (2000) observation that students may endorse growth-oriented beliefs while simultaneously drawing on fixed-mindset explanations of performance. Students in this study often reported that their math ability could change, yet attributed this change primarily to instructor influence rather than their own strategic effort. This pattern parallels the “Professor Impact” theme identified in our data and resonates with Boaler’s (2013) and Stevenson et al.’s (1993) concerns about persistent cultural narratives of mathematical talent. It also aligns with He and Zhang’s (2024) evidence that the strength of GM and learning self-efficacy links varies across cultural orientations, weakening in hierarchical contexts. Within this study, students’ reliance on instructors rather than self-regulation may similarly reflect classroom hierarchies that shape how growth-oriented beliefs are enacted.

The finding that students across both treatment and control conditions were willing to use SRL resources independently—often favoring online tools over faculty support—adds complexity to Zimmerman’s (2000) model of SRL. Students demonstrated behavioral engagement with self-regulatory tools but did not consistently connect these behaviors to GM reasoning, suggesting a disconnect between beliefs and practices. This aligns with Marchis’s (2012) and Fauzi and Widjajanti’s (2018) emphasis on the need for explicit metacognitive framing in mathematical SRL instruction. It also raises questions about modality preferences: whether students who favor out-of-class practice or video-based resources are more likely to adopt classroom-integrated strategy instruction when it aligns with their preferred modes of engagement. In this respect, Lee and Kwon (2023) provide a useful post-pandemic perspective, showing that purposeful use of technology in blended designs can enhance motivation and well-being, particularly when coupled with community building and strong instructor presence.

Finally, student reports that some activities resembled “busy work” reflect challenges of alignment between strategy instruction and perceived task relevance. Weinstein et al. (2000) emphasized that learning strategies are most effective when clearly connected to task demands, and a lack of this alignment may partly explain the limited impact reported here. In addition, Wang et al. (2023) highlight that SRL is multidimensional, with motivational, cognitive, metacognitive, and social dimensions. The present findings reinforce the importance of recognizing these dimensions when evaluating how students perceive and take up explicit learning-strategy interventions.

### Limitations and Future Research

The study’s methodological choices introduce several limitations that also point toward potential research directions. First, missing data were handled using listwise deletion, which reduced the effective sample size. Future studies could apply multiple imputation to preserve statistical power and assess the robustness of findings. Second, potential instructor effects could not be modeled due to small within-instructor subsamples. Subsequent research should use larger samples or employ hierarchical modeling to examine how variations in instructional style moderate intervention outcomes. Third, the overrepresentation of treatment-condition students in the qualitative data may have biased thematic saturation; more balanced sampling would strengthen cross-condition comparisons. Fourth, the focus on short-term, end-of-course outcomes leaves open the question of whether GM and SRL effects might accumulate over multiple semesters. Future research could, therefore, track students longitudinally, employ retrospective pretest–posttest designs (Little et al., 2020) to better capture self-reported change, and examine potential mediators such as course participation and engagement with online tools.

## 5. Conclusions

This study investigated whether integrating explicit instruction in cognitive, metacognitive, and management learning strategies into gateway mathematics courses at an HBCU could strengthen students’ math growth mindset (GM), self-regulated learning (SRL), and academic performance. Quantitative analyses showed no statistically significant short-term improvements in GM or SRL. While both were positively, though nonsignificantly, associated with performance, the intervention itself was linked to a modest decline in performance when controlling for mediators. This suggests possible implementation or alignment issues that warrant further investigation. Qualitative evidence further suggested that students’ beliefs about math ability remained shaped by instructor influence and entrenched cultural narratives of talent, underscoring the challenges of shifting mindset and self-regulation within a single semester.

These findings highlight several implications for research and practice. First, altering students’ beliefs and behaviors likely requires sustained, multi-semester exposure to strategy-based instruction rather than one-semester interventions. Second, embedding learning strategies into course assessments and authentic problem-solving tasks is essential to enhance their perceived relevance and uptake. Third, strategy instruction should be framed metacognitively, emphasizing students’ agency in applying strategies so that SRL becomes internalized rather than dependent on instructor style. Finally, at minority-serving institutions, coupling strategy instruction with culturally responsive pedagogy and faculty engagement is especially important for addressing equity gaps.

Taken together, the results suggest that strategy-based interventions in postsecondary math should not only teach specific strategies but also reframe how students view effort, persistence, and their own capacity to succeed. Embedding learning strategies as a structural and culturally responsive component of instruction offers a promising pathway for strengthening GM, SRL, and persistence in mathematics, particularly among underrepresented students.

## Figures and Tables

**Table 1:** Components of the learning-strategies instruction intervention.

Component	Description	Focus
Discussion Boards (5 total)	Video reflections and peer responses on learning strategies, time/test management, and study habits.	Metacognitive, Management
Class Discussions (5 total)	Instructor-led discussions on effort, elaboration, self-testing, and exam strategies.	Cognitive, Metacognitive, Effort Management
Study Plans & Self-Assessments	Use of Pearson tools for personalized review before tests.	Planning, Self-monitoring
Study Calendars	Students created and submitted weekly study schedules.	Time Management
Peer Tutor Presentations	Live/pre-recorded presentations by former students sharing successful math learning strategies.	Modeling, Strategy Awareness

**Table 2:** Characteristics of the sample participants by their role in the study.

Variable	Control: *n* (%)	Treatment: *n* (%)
Total *n*	230	255
Gender: Female	141 (61.30%)	178 (69.80%)
STEM: Yes	130 (56.52%)	127 (49.80%)
PELL: Yes	210 (91.30%)	226 (88.63%)
Residency: Out-of-State	116 (50.43%)	120 (47.06%)
Rural: Yes	35 (15.22%)	37 (14.51%)
Classification: Freshman	160 (69.60%)	163 (63.90%)
Classification: Sophomore	59 (25.65%)	76 (29.80%)
Classification: Junior	11 (4.78%)	16 (6.27%)
Course: College Alg. I	76 (33.00%)	85 (33.30%)
Course: College Alg. II	49 (21.30%)	59 (23.14%)
Course: Calculus I	68 (29.57%)	71 (27.84%)
Course: Calculus II	37 (16.09%)	40 (15.69%)
Course Grade: A	31 (13.50%)	49 (19.20%)
Course Grade: B	51 (22.17%)	70 (27.45%)
Course Grade: C	79 (34.35%)	82 (32.16%)
Course Grade: D	24 (10.43%)	19 (7.45%)
Course Grade: F	45 (19.57%)	35 (13.73%)

**Table 3:** Psychometric statistics for the different study scales.

Scale	Pre-Survey		Post-Survey	
	RMSR	Omega	RMSR	Omega
Math Mindset	0.003	0.69	0.002	0.73
SRL	0.057	0.84	0.063	0.83
Math Identity	0.015	0.88	0.015	0.89
Gender Identity: Female	0.017	0.52	0.003	0.59
Gender Identity: Male	0.012	0.58	0.018	0.49
Racial Identity: Black	0.015	0.56	0.023	0.57
Racial Identity: Non-Black	0.015	0.89	0.032	0.83

**Table 4:** Summary statistics of response variables by intervention.

Variable	Control			Treatment		
	Pre	Post	Diff	Pre	Post	Diff
	Mean (*SD*)	Mean (*SD*)	Mean (*SD*)	Mean (*SD*)	Mean (*SD*)	Mean (*SD*)
Mindset	4.47 (0.90)	4.27 (0.96)	−0.20 (0.87)	4.42 (0.98)	4.25 (1.04)	−0.18 (1.03)
SRL-1	3.43 (0.67)	3.45 (0.63)	0.03 (0.53)	3.41 (0.66)	3.45 (0.66)	0.04 (0.50)
SRL-2	3.89 (0.57)	3.74 (0.64)	−0.16 (0.60)	3.88 (0.60)	3.65 (0.72)	−0.21 (0.70)
Performance	38.90 (17.18)	63.53 (20.66)	23.86 (20.39)	39.95 (16.87)	62.28 (21.60)	21.54 (21.66)

**Table 5:** Path analysis estimates (Models 1–3).

Path	Reg. Eq. (Source) ^1^	Estimate (β)	SE ^2^	95% CI	*p*-Value
a_1_: T → $GM_{post}$	Model 1	−0.0457	0.0869	[−0.2164, 0.2151]	0.5994
a_2_: T → $SRL1_{post}$	Model 2	−0.0056	0.0483	[−0.1006, 0.0895]	0.9079
a_2’_: T → $SRL2_{post}$	Model 2′	−0.0819	0.0607	[−0.2011, 0.0374]	0.1780
b_1_: ${GM}_{post}$ → $Y_{post}$ (controlling SRL)	Model 3	0.8873	0.9793	[−1.0393, 2.8138]	0.3656
b_2_: $SRL1_{post}$ → $Y_{post}$ (controlling GM)	Model 3	0.3672	1.7327	[−3.0417, 3.7760]	0.8323
b_2’_: $SRL1_{post}$ → $Y_{post}$ (controlling GM)	Model 3	2.3080	1.4087	[−0.4633, 5.0792]	0.1023
c′: Direct effect T → $Y_{post}$ (controlling mediators)	Model 3	−3.8298	1.5781	[−6.9344, −0.7252]	0.0158

^1^ All models control for baseline covariates: $Y_{pre}$, ${GM}_{pre}$, ${SRL}_{pre}$, identity covariates (math, gender, racial identities), and other pre-specified covariates **X**. ^2^ SE = classical OLS standard error. Robust SEs (type = “HC3”) were almost identical to the OLS SEs and hence were omitted.

**Table 6:** Decomposition of treatment effect: indirect effects, direct effect, and total effect.

Effect	Point Estimate ^1^	SE (Boot) ^2^	95% CI (BCa) ^2^
Indirect via GM (a_1_ × b_1_)	−0.0405	0.1263	[−0.4881, 0.l003]
Indirect via SRL1 (a_2_ × b_2_)	−0.0021	0.0862	[−0.2182, 0.1624]
Indirect via SRL2 (a_2’_ × b_2’_)	−0.1889	0.2041	[−0.8790, 0.0342]
**Total indirect (sum)**	−0.2315	0.2697	[−0.9840, 0.1429]
Direct effect (c′)	−3.8298	1.5557	[−6.7280, −0.5830]
**Total effect (c′ + indirect)**	−4.0613	1.5334	[−6.9450, −0.947]
% mediated (total indirect/total)	5.70%	—	—

^1^ All models control for baseline covariates: $Y_{pre}$, ${GM}_{pre}$, ${SRL}_{pre}$, identity covariates, and other pre-specified X. ^2^ Bootstrap SE and confidence intervals (BCa) used R = 2000 replications.

**Table 7:** Estimates of regression coefficients (standard errors) from regression models 1–3, with the response variable shown in the column and explanatory variables shown in the rows.

Response Variable	Mindset Post	SRL-1 Post	SRL-2 Post	Performance Post
Explanatory Variable ^1^	Estimate (S.E.) ^3^	Estimate (S.E.)	Estimate (S.E.)	Estimate (S.E.)
Role: Treatment	−0.0456 (0.0869)	−0.0056 (0.0483)	−0.0819 (0.0607)	−3.8298 (1.5781) *
Score Pre	−0.0038 (0.0032)	0.0001 (0.0018)	0.0002 (0.0022)	0.1182 (0.0565) *
Mindset Pre	0.4641 (0.0502) ***	0.0064 (0.0280)	0.0587 (0.0352) .	−1.6377 (1.0004)
SRL-1 Pre	−0.1107 (0.0752)	0.6558 (0.0483) ***	−0.0040 (0.0526)	−1.9036 (1.7327)
SRL-2 Pre	0.2964 (0.0864) ***	0.0485 (0.0484)	0.4591 (0.0607) ***	−2.8999 (1.6870) .
Mindset Post	—	—	—	0.8873 (0.9793)
SRL-1 Post	—	—	—	0.3672 (1.7327)
SRL-2 Post	—	—	—	2.3080 (1.4087)
Gen Identity: Reflection	−0.0515 (0.0374)	0.0475 (0.0208) *	−0.0012 (0.0261)	−0.6093 (0.7268)
Gen Identity: Centrality	0.0379 (0.0372)	0.0221 (0.0206)	0.0451 (0.0259) .	0.4192 (0.6746)
Racial Identity	0.0260 (0.0565)	−0.0140 (0.0314)	−0.0062 (0.0395)	−0.1304 (1.0548)
Math Iden: Competency	0.1711 (0.0929) .	0.0779 (0.0520)	0.0888 (0.0653)	−0.7478 (1.7304)
Math Iden: Recognition	0.0716 (0.0614)	−0.0206 (0.0341)	−0.0874 (0.0428) *	1.6403 (1.1381)
Math Iden: Interest	−0.1153 (0.0520) *	−0.0423 (0.0289)	0.0353 (0.0362)	−0.1840 (0.9689)
Gender: Male	−0.03 58 (0.1055)	0.0910 (0.0592)	0.1254 (0.0742) .	0.7765 (1.9251)
STEM: Yes	−0.1809 (01021) .	−0.0936 (0.0567) .	−0.1650 (0.0712) *	−0.7672 (1.8552)
Letter Grade: B	−0.1821 (0.1376)	−0.0584 (0.0758)	−0.1878 (0.0962) .	−8.1689 (2.5700) **
Letter Grade: C	−0.3057 (0.1361) *	−0.1352 (0.0758) .	−0.3369 (0.0952) ***	−18.1729 (2.6371) ***
Letter Grade: D	−0.3230 (0.1915) .	−0.1986 (0.1069) .	−0.2360 (0.1341) .	−23.0977 (3.5110) ***
Letter Grade: F	−0.2443 (0.1761)	−0.1931 (0.0980) *	−0.2561 (0.1230) *	−27.6652 (3.5110) ***
PELL Eligible: Yes	0.0787 (0.1602)	0.1150 (0.0887)	0.0564 (0.1113)	6.3218 (2.8939) *
Residency: Out-of-State	0.0420 (0.0890)	−0.0340 (0.0494)	−0.0509 (0.0620)	1.8657 (1.6322)
Classification: Sophomore ^2^	0.1743 (0.2102)	−0.0153 (0.0583)	−0.0143 (0.1496)	−1.1932 (1.9165)
Classification: Junior	0.1283 (0.1051)	0.0919 (0.1192)	−0.0492 (0.0732)	0.6121 (3.9602)
Course: Algebra II	−0.1584 (0.1336)	0.0262 (0.0742)	0.0299 (0.0932)	−5.9890 (2.3230) *
Course: Calculus I	0.0322 (0.1554)	0.0875 (0.0869)	0.0346 (1091)	−18.8652 (3.0920) ***
Course: Calculus II	0.0840 (0.1705)	0.1684 (0.0948) .	0.1551 (0.1190)	5.9568 (3.0760) .
Attendance	0.0032 (0.0042)	0.0004 (0.0023)	0.0052 (0.0029) .	−0.1389 (0.0784) .
$\mathrm{Adjusted}\;R^{2}$	0.2958	0.4697	0.2559	0.5478
*n*	407	401	401	341

^1^ Reference category is “Control” for *Role*, “Female” for *Gender*, “No” for *STEM* and *PELL*, “A” for *Letter Grade*, “In-State” for *Residency*, “Freshman” for *Classification*, and “Algebra I” for *Course*. ^2^ Only one student had a “Senior” classification and, thus, was omitted from the analysis. ^3^ Significance codes: ‘***’ ≡ *p*-value < 0.001; ‘**’ ≡ *p*-value < 0.01; ‘*’ ≡ *p*-value < 0.05; ‘·’ ≡ *p*-value < 0.1.

**Table 8:** Qualitative study participants by course and condition.

Focus Group/Interview	Course	Condition	Participants
1	Math 103 Algebra I	Control	3
2	Math 103 Algebra I	Control	4
3	Math 103 Algebra I	Treatment	2
4	Math 104 Algebra II	Control	3
5	Math 104 Algebra II	Treatment	6
6	Math 131 Calculus I	Control	3
7	Math 131 Calculus I	Treatment	5
8	Math 131 Calculus I	Treatment	1
9	Math 131 Calculus I	Treatment	2
10	Math 132 Calculus II	Control	4
11	Math 132 Calculus II	Treatment	1
12	Math 132 Calculus II	Treatment	3

**Table 9:** Codes for a sample of focus group questions.

Code	Protocol Question
Course Experience	Can you describe what your experiences were like in the Math course?
Course Like	What did you like [about the Math course]?
Course Dislike	What did you dislike [about the Math course]?
Course Structure	How did the structure/format of this course compare or differ with other math courses you have completed?
Course Discussion Questions	Did you have discussion boards in this course?
Course Learning Strategies	How would you describe your ability in this class?
Course Motivation	How would you describe your motivation in this class?

## Data Availability

The data presented in this study are available on request from the corresponding author. The data are not publicly available due to privacy and IRB protocol restrictions.

## Appendix A

***Learning-strategy instruction in introductory Math courses and expected outcomes.***

**Course Topic(s) (Module)**	**Learning Objective**	**Integrated Learning Strategy**	**Integration Method(s)**	**Expected Outcomes**
**MATH103: College Algebra I**				
- Algebra Essentials - Rational Expressions, Radicals, Linear & Quadratic Equations, & Complex #s	Students will apply quantitative and mathematical reasoning skills in examining, evaluating, and solving problems involving order of operation, factoring, solving equations, and functions.	**Metacognitive** (Self-Testing)	In- and out-of-class self-test quizzes	Students will learn to **use self-testing to assess** their understanding of how to simplify, factor, and perform basic operations on algebraic expressions, including polynomials, rational and radical expressions, and complex fractions/numbers.
	**Metacognitive** (Adaptation of Learning Approach)		Class presentations/discussions	Students will learn to **choose the most direct/efficient method** to solve linear, absolute value, rational, radical, and quadratic equations by symbolic methods.
- Equations and Inequalities	Students will evaluate quantitative information using a variety of methods.	**Metacognitive** (Self-Testing)	In- and out-of-class self-test quizzes	Students will be able to **self-assess** their comprehension of how to solve various types of equations and inequalities.
- Graphs and Functions	Students will organize, analyze, present, and communicate quantitative information in diverse ways.	**Cognitive** (Elaboration)	Class discuss/discuss-board assign	Students will learn to **use elaboration to identify, summarize, and connect between** key features of a graph (intercept; slope; intervals where function is increasing/decreasing/+/−; relative max/min; symmetry; and end behavior).
**MATH104: College Algebra II**				
- Polynomials - Exponential & Log Functions	Students will apply mathematical reasoning skills to examine, evaluate, and solve problems about polynomials, and exponential and logarithmic functions.	**Metacognitive** (Self-Testing)	In- and out-of-class self-test quizzes	Students will learn to **self-evaluate their competence to work with** polynomial, exponential, and logarithmic functions.
	**Metacognitive** (Adaptation of Learning Approach)		Class presentations/discussions	Students will learn to **modify their solution approaches to efficiently** find the zeroes of polynomial, exponential, and logarithmic functions.
	**Cognitive** (Elaboration)		Class discussions/discussion-board assign	Students will use **elaboration to distinguish** between the characteristics of polynomial, exponential and logarithmic functions/graphs; and use **paraphrasing** to write their own interpretations of these functions/graphs.
- Systems of Equations and Matrices	Students will organize and evaluate quantitative information using a variety of methods.	**Metacognitive** (Self-Testing)	In- and out-of-class self-test quizzes	Students will learn to **self-test** their ability to work with systems of equations and matrices.
	**Metacognitive** (Adaptation of Learning Approach)		Class presentations/discussions	Students will learn to **choose an efficient method** (e.g., elimination, substitution, etc.) to solve linear equations in 2 or 3 variables as well as nonlinear systems of eqs.
- Trigonometric Functions - Trigonometric Identities & Applications	Students will apply mathematical reasoning skills to examine, evaluate, and solve problems about trigonometric functions and learn how to apply these functions in different situations.	**Cognitive** (Elaboration)	Class discussions/discussion-board assign	Students will use elaboration to **summarize** the features of trigonometric functions and identities; **identify** trigonometric functions by right triangle for any angle; and **categorize** the various trigonometric identities (identities of co-functions, identities of negative angles, etc.).
**MATH131: Calculus I**				
- Functions	Students will accurately communicate mathematical information in graphical, verbal, or equation forms.	**Cognitive** (Elaboration)	Class discussions/ discussion-board assignments	Students will learn to **use elaboration** (summarization and paraphrasing) to enhance their conception of the mathematical notation for various sets of real numbers, and the descriptions of lines, circles, and other basic sets in the coordinate plane.
- Limits	Students will develop mathematical skills to formulate the instantaneous rate of change of a function.	**Metacognitive** (Adaptation of Learning Approach)	Class presentations/discussions	Students will learn to **choose and adapt** an efficient way (graphical, numerical, or algebraic) to evaluate limits of different kinds of functions.
	**Metacognitive** (Self-Testing)		In- and out-of-class self-test quizzes	Students will **self-assess** their understanding of the concept of continuity and their ability to examine the continuity of functions at a point or on an interval.
- Derivatives	Students will learn the definition of the derivative and be fluent with the concept of slope of a tangent line and instantaneous rate of change of a function.	**Cognitive** (Elaboration)	Class discussions/discussion-board assignments	Students will use **paraphrasing** to make their own intuitive interpretation of derivatives and will learn to use **summarization** to organize the derivative rules and **connect** these rules with their applications.
	**Metacognitive** (Adaptation of Learning Approach)		Class presentations/discussions	Students will learn to **adapt** their approach when computing derivatives of different kinds of functions via introducing them to the different ways in which derivatives arise.
- Integration	Students will learn the connection between derivatives and integrations.	**Metacognitive** (Self-Testing)	In- and out-of-class self-test quizzes	Students will **self-evaluate** their ability to apply integration techniques to compute areas under curves, surface areas, and volumes.
	**Cognitive** (Elaboration)		Class discussions/discussion-board assignments	Students will **use elaboration** to draw meaningful connections between derivatives and integrations.
**MATH132: Calculus II**				
- Techniques & Applications of Integration	Students will accurately evaluate integrals using a variety of methods.	Metacognitive (Self-Testing)	In- and out-of-class self-test quizzes	Students will **practice self-testing** to evaluate and enhance their understanding of basic techniques for evaluating integrals (e.g., slice-and-sum strategy) and geometric applications of integration.
	**Metacognitive** (Adaptation of Learning Approach)		Class presentations/discuss	Students will learn to **select and adapt** the most efficient/appropriate method to evaluate any given integral.
- Sequences and Infinite Series - Power Series	Students will tell the distinction between a sequence and an infinite series, apply quantitative and mathematical reasoning to solve problems using series.	**Cognitive** (Elaboration)	Class discussions/discussion-board assignments	Students will **use elaboration** to **distinguish** between sequences and series, to **summarize and group** the different methods for evaluating limits of sequences and determining convergence of series, and to **connect** betw. concepts of limits and convergence.
	**Metacognitive** (Self-Testing)		In- and out-of-class self-test quizzes	Students will **practice self-testing** to assess and consolidate their mastery of power series and their properties and applications for approximating functions.
- Parametric and Polar Curves	Students will efficiently communicate quantitative or mathematical information in graphical or equation forms.	**Metacognitive** (Adaptation of Learning Approach)	Class presentations/discussions	Students will learn to **adapt their learning approach** via introducing them to the various alternative ways for generating curves and representing functions.

## Appendix B

***Students’ Math Mindset Survey Questions***

**To what extent do you disagree or agree with each of the following statements?**

Strongly disagree	Disagree	Somewhat disagree	Somewhat agree	Agree	Strongly agree

1. Anyone can be good at math if they work hard at it. (**Mindset**, Reverse)—Omitted
2. You have a certain amount of math intelligence, and you can’t really do much to change it. (**Mindset**)
3. There are limits to how much people can improve their basic math ability. (**Mindset**)
4. Some kids can never do well in math, even if they try hard. (**Mindset**)

## Appendix C

***Students’ Self-Regulated Learning Survey Questions***

For each statement, please check ONE circle to indicate HOW OFTEN you do each of these things when doing homework for MATH or studying for MATH tests.

Almost Never	Not Very Often	Somewhat Often	Very Often	Almost Always

Three subscales: SRL1: Managing environment and behavior; SRL2: Maladaptive regulatory behavior; and SRL3: Seeking and learning information.

1. I make sure no one disturbs me when I study. (**SRL1**)
2. I try to study in a quiet place. (**SRL1**)
3. I think about the types of questions that might be on a test. (**SRL3**)
4. I ask my math teacher about the topics that will be on upcoming tests. (**SRL3**)
5. I rely on my math class notes to study. (**SRL3**)
6. I study hard even when there are more fun things to do at home. (**SRL1**)
7. I quiz myself to see how much I am learning during studying. (**SRL1**)
8. I make a schedule to help me organize my study time. (**SRL1**)
9. I use binders or folders to organize my math study materials. (**SRL1**)—Omitted
10. I lose important math worksheets or materials. (**SRL2**)
11. I avoid going to extra-help sessions in math. (**SRL2**)—Omitted
12. I wait to the last minute to study for math tests. (**SRL2**)
13. I try to forget about the topics that I have trouble learning. (**SRL2**)
14. I try to see how my notes from my math class relate to things I already know. (**SRL3**)
15. I try to identify the format of upcoming math tests (e.g., multi-choice, essay, test length). (**SRL3**)
16. I try to study in a place that has no distractions (e.g., noise, people talking). (**SRL1**)
17. I ask my teacher questions when I do not understand something. (**SRL3**)
18. I make pictures or drawings to help me learn math concepts. (**SRL3**)—Omitted
19. I give up or quit when I do not understand something. (**SRL2**)
20. I forget to bring home my math materials when I need to study. (**SRL2**)
21. I tell myself exactly what I want to accomplish during studying. (**SRL1**)—Omitted
22. I look over my homework assignments if I don’t understand something. (**SRL3**)
23. I avoid asking questions in class about things I don’t understand. (**SRL2**)
24. I tell myself to keep trying when I can’t learn a topic or idea. (**SRL1**)
25. I carefully organize my study materials, so I don’t lose them. (**SRL1**)
26. I let my friends interrupt me when I am studying. (**SRL2**)
27. I think about how to best study before I begin studying. (**SRL1**)
28. I finish all of my studying before I play video games or go out with my friends. (**SRL1**)

## Appendix D

***Students’ Math Identity Survey Questions***

To what extent do you disagree or agree with each of the following statements?

Strongly disagree	Disagree	Not sure	Agree	Strongly agree

1. I am confident that I can understand math in class. (**Competency**)
2. I am confident that I can understand math outside of class. (**Competency**)
3. I can do well on exams in math. (**Competency**)
4. I understand concepts I have studied in math. (**Competency**)
5. Others ask me for help in math. (**Recognition**)
6. I can overcome setbacks in math. (**Competency**)
7. My parents/relatives/friends see me as a math person. (**Recognition**)
8. My math teachers see me as a math person. (**Recognition**)
9. I am interested in learning more about math. (**Interest**)
10. I enjoy learning math. (**Interest**)

## Appendix E

***Students’ Gender Identity Survey Questions***

The questions in this section try to measure your gender identity. There are no right or wrong answers. Your honest opinions and answers will help us to improve math education.

To what extent do you disagree or agree with each of the following statements?

Strongly disagree (1)	(2)	(3)	Neutral (4)	(5)	(6)	Strongly agree (7)

Two subscales: Reflection & Centrality.

Females

1. Overall, being a woman has very little to do with how I feel about myself. **Reverse**—Omitted
2. In general, being a woman is an important part of my self-image. (**Reflection**)
3. My destiny is tied to the destiny of other women. (**Centrality**)
4. Being a woman is unimportant to my sense of what kind of person I am. **Reverse**—Omitted
5. I have a strong sense of belonging to women as a group. (**Centrality**)
6. I have a strong attachment to other women. (**Centrality**)
7. Being a woman is an important reflection of who I am. (**Reflection**)
8. Being a woman is not a major factor in my social relationships. **Reverse**—Omitted

Males

1. Overall, being a man has very little to do with how I feel about myself. **Reverse**—Omitted
2. In general, being a man is an important part of my self-image. (**Reflection**)
3. My destiny is tied to the destiny of other men. (**Centrality**)
4. Being a man is unimportant to my sense of what kind of person I am. **Reverse**—Omitted
5. I have a strong sense of belonging to men as a group. (**Centrality**)
6. I have a strong attachment to other men. (**Centrality**)
7. Being a man is an important reflection of who I am. (**Reflection**)
8. Being a man is not a major factor in my social relationships. **Reverse**—Omitted

## Appendix F

***Students’ Racial Identity Survey Questions***

In this country, people come from a lot of different cultures and there are many different words to describe the different backgrounds or ethnic groups that people come from. Some examples of the names of ethnic groups are Hispanic, Black, Asian-American, Native American, Irish-American, and White. The following questions are about your ethnicity or your ethnic group and how you feel about it or react to it.

To what extent do you disagree or agree with each of the following statements?

Strongly disagree (1)	(2)	(3)	Neutral (4)	(5)	(6)	Strongly agree (7)

**Three subscales: Centrality, Private Regard, and Public Regard**.

**Black (African American)**

1. Overall, being Black has very little to do with how I feel about myself. (**Centrality**)
2. In general, being Black is an important part of my self-image. (**Centrality**)
3. My destiny is tied to the destiny of other Black people. (**Centrality**)
4. Being Black is unimportant to my sense of what kind of person I am. **Reverse**—Omitted
5. I have a strong sense of belonging to Black people. (**Centrality**)
6. I have a strong attachment to other Black people. (**Centrality**)
7. Being Black is an important reflection of who I am. (**Centrality**)
8. Being Black is not a major factor in my social relationships. **Reverse**—Omitted
9. I feel good about Black people. (**Private Regard**)
10. I am happy that I am Black. (**Private Regard**)
11. I feel that Blacks have made major accomplishments and advancements. (**Private Regard**)
12. I am proud to be Black. (**Private Regard**)
13. I feel that the Black community has made valuable contributions to this society. (**Private Regard**)
14. I often regret that I am Black. **Reverse**—Omitted
15. Overall, Black people are considered good by others. (**Public Regard**)
16. In general, others respect Black people. (**Public Regard**)
17. Most people consider Black people, on the average, to be more ineffective than other racial groups. **Reverse**—Omitted
18. Black people are not respected by the broader society. **Reverse**—Omitted
19. In general, other groups view Black people in a positive manner. (**Public Regard**)
20. Society views Black people as an asset. (**Public Regard**)

**Non-Black**

The following questions are about your ethnicity or your ethnic group and how you feel about it or react to it.

To what extent do you disagree or agree with each of the following statements?

Strongly disagree	Disagree	Neutral	Agree	Strongly agree

1. I have spent time trying to find out more about my ethnic group, such as its history, traditions, and customs. (**Exploration**)
2. I have a strong sense of belonging to my own ethnic group. (**Commitment**)
3. I understand pretty well what my ethnic group membership means to me. (**Commitment**)
4. I have often done things that will help me understand my ethnic background better. (**Exploration**)
5. I have often talked to other people in order to learn more about my ethnic group. (**Exploration**)
6. I feel a strong attachment towards my own ethnic group. (**Commitment**)

## Appendix G

***Students’ Focus Group Protocol***

**General Questions**

1. Please briefly introduce yourself, including your name, cohort and major.
2. What do you like about math?
  - When are you most engaged in math (e.g., context, activity, lecture, instructor)?
3. When you are learning a new math concept, what is your ideal way of learning?
4. What do you dislike about math.
5. How would you describe your abilities in math?
  - What factors influence these abilities?
6. To what degree have your abilities changed (or not changed) over time?
7. Before this course, how did you usually study for exams or prepare for class?

**Course Specific Questions**

1. Can you describe what your experiences were like in the MATH course?
  - What did you like?
  - What did you dislike?
  - How did the structure/format of this course compare or differ with other math courses you have completed?
    - Did you have discussion boards in this course?
2. How would you describe your motivation in this class?
  - What factors influenced your motivation?
3. How would you describe your ability in this class?
  - What factors influenced your ability?
  - Do you believe your math ability has or can change or grow?
    - Did you always think this way? If not, what has made you think differently?
4. How much effort do you put into completing tasks for this class each week inside and outside of class?
  - What types of tasks did you do to help you learn math, e.g., did you quiz yourself often? What other things did you do?
  - What factors influence the amount of effort you put into the course?
5. Can you describe any challenges or difficulties you experienced?
6. When you faced these challenges/difficulties what strategies or resources did you use to overcome them?
7. Do you think of yourself as a math person? (Someone who likes math or to whom math comes easily)?
  - Did you think this way before you took this course?
8. Do you think others think of you as a math person?
  - Did they think this way before you took this course?
9. Do you believe your race/ethnicity has affected your experiences with math before this course? If so, how so? After this course? If so, how so?
10. Do you believe your gender has affected your experiences with math before this course? If so, how so? After this course? If so, how so?
11. Is there anything else you would like to add?
